# Extracellular RNA in melanoma: Advances, challenges, and opportunities

**DOI:** 10.3389/fcell.2023.1141543

**Published:** 2023-05-04

**Authors:** Zhouxiao Li, Yiyang Gao, Yang Cao, Feifan He, Runyi Jiang, Hanyuan Liu, Hongzhou Cai, Tao Zan

**Affiliations:** ^1^ Department of Plastic and Reconstructive Surgery, Ninth People’s Hospital, Shanghai Jiao Tong University School of Medicine, Shanghai, China; ^2^ Department of Radiology, The Fourth School of Clinical Medicine, Nanjing Medical University, Nanjing, Jiangsu, China; ^3^ Department of Orthopaedic Oncology, Spinal Tumor Center, Second Affiliated Hospital of Naval Medical University, Shanghai, China; ^4^ Department of General Surgery, Nanjing First Hospital, Nanjing Medical University, Nanjing, Jiangsu, China; ^5^ Department of Urology, Jiangsu Cancer Hospital and The Affiliated Cancer Hospital of Nanjing Medical University and Jiangsu Institute of Cancer Research, Nanjing, Jiangsu, China

**Keywords:** melanoma, RNA, exosomes, dermatology, tumor environment (TME)

## Abstract

Melanoma, a malignant mass lesion that originates in melanocytes and has a high rate of malignancy, metastasis, and mortality, is defined by these characteristics. Malignant melanoma is a kind of highly malignant tumor that produces melanin and has a high mortality rate. Its incidence accounts for 1%–3% of all malignant tumors and shows an obvious upward trend. The discovery of biomolecules for the diagnosis and treatment of malignant melanoma has important application value. So far, the exact molecular mechanism of melanoma development relevant signal pathway still remains unclear. According to previous studies, extracellular RNAs (exRNAs) have been implicated in tumorigenesis and spread of melanoma. They can influence the proliferation, invasion and metastasis of melanoma by controlling the expression of target genes and can also influence tumor progression by participating in signal transduction mechanisms. Therefore, understanding the relationship between exRNA and malignant melanoma and targeting therapy is of positive significance for its prevention and treatment. In this review, we did an analysis of extracellular vesicles of melanoma which focused on the role of exRNAs (lncRNAs, miRNAs, and mRNAs) and identifies several potential therapeutic targets. In addition, we discuss the typical signaling pathways involved in exRNAs, advances in exRNA detection and how they affect the tumor immune microenvironment in melanoma.

## 1Introduction

A common form of cancer is skin cancer, which accounts for one-third of all cancers worldwide (Hay et al., 2014; Guy et al., 2015a; Guy et al., 2015b). Cancers of the skin generally fall into three categories: basal cell carcinoma, squamous cell carcinoma, and malignant melanoma. Melanoma is one of the most common types of skin cancer, which have increased dramatically over the past decade (Gloster and Neal, 2006; Erdei and Torres, 2010). Among the most common genes implicated in the development of melanoma are BRAF, CDKN2A, NRAS, and TP53. Among them, BRAF is highly correlated with exRNAs. It is the key regulator of RAS-RAF-MEK-ERK pathway, which is well known as MAPK (mitogen-activated protein kinase)/ERK (extracellular signal-regulated kinase ERK). The activation of BRAF triggers the phosphorylation of the ERK, which is common in variant cancers. Using the V600E mutation as an example and discussing its effects, we present our findings in this manuscript (Mao et al., 2021).

Extracellularly released RNA species, collectively known as extracellular RNAs (exRNAs), have been demonstrated in several previous studies. A significant component of cancer-related alterations in the circulating biomaterial is extracellular RNA molecules (exRNAs). ExRNA circulates in extracellular fluid after separating from the intracellular environment. ExRNAs are typically encapsulated in extracellular vesicles (EVs) or form complexes with proteins (Gloster and Neal, 2006). In addition to providing valuable biomarkers for the development of liquid biopsy-based assays, they are also detectable in blood, urine, and saliva. MicroRNAs, small interfering RNAs, small nucleolar RNAs, and long stranded non-coding RNAs are among the extracellular RNAs capable of regulating mRNAs. The most extensively studied miRNAs, with over 2000 identified to date, have been the most extensively studied. Various small RNAs are released into the extracellular environment as cargoes of EV, specifically known as exosomes, as part of EV. Having reviewed these findings, it is now apparent that extracellular RNA communication has significant implications for the fields of biology, disease, and medicine (Mugoni et al., 2022). Furthermore, ExRNAs play a crucial role in melanoma, where melanoma cells produce numerous types of EVs capable of communicate with each other and signal within the tumor microenvironment. An exosome, a microvesicle, or apoptotic body is a subpopulation of an EV that carries genetic material and protein. According to the sequence analysis, EV subpopulations contain numerous and distinct non-coding RNAs, including microRNAs, mitochondria-associated tRNAs, micronucleus RNAs, micronucleus RNAs, Ro-associated Y-RNAs, Vault RNAs, and Y-RNAs. Several miRNAs expressed in exosomes are not present in benign nevi, but are expressed in melanomas. This is supported by a comparison between exosomal miRNAs and those present in melanoma samples. Lunavat et al. (2015). As well as reviewing exRNAs and possible therapeutic targets in EVs of melanoma, this paper explains their role in EVs. Besides, recent advances in techniques for detecting exRNAs are discussed as they relate to the immune microenvironment of melanoma.

## 2 Biogenesis and structure of extracellular vesicles (EVs)

In cell-derived vesicles, EVs transport a variety of cargoes outside of the cell. It can be argued that extracellular vesicles serve as vehicles for intercellular communication or as “homing pigeons” since their molecules of content (proteins, non-coding RNAs, miRNAs, DNA, *etc.*) are delivered to neighbors and distant cells. A recent study has shown that the EVs secreted by cancer cells differ in composition and quantity from those secreted by normal cells. They may influence the tumor microenvironment and contribute to tumorigenesis (Cesi et al., 2016). In several cancers including melanoma, EVs are released in high amounts by cancer cell lines and patients (Logozzi et al., 2009; King et al., 2012).

**FIGURE 1:**
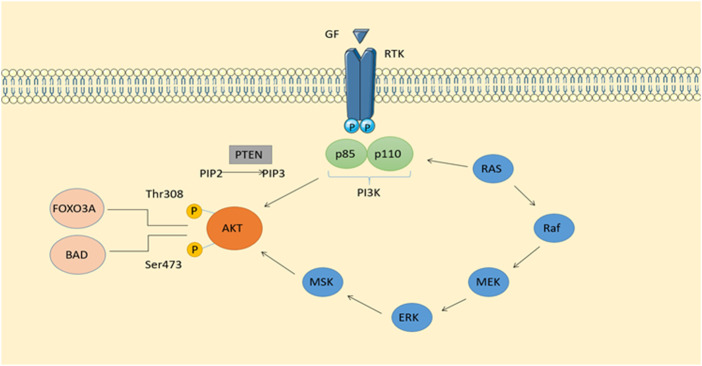
Phosphatidylinositol-related signaling pathways and signaling pathways derived from RTKs make up the classical intracellular signaling pathway PI3K-AKT. In addition to stimulating insulin and maintaining cell viability, it also plays an important role in cell growth. Activation of calcium-regulated protein kinase II, which activates Ras, the protein kinase of ERKl2, is possible when calcium levels are high.

In latest studies, mesenchymal stem cells have been shown to secrete more microvesicles under hypoxic conditions. Zhang et al. reported that mesenchymal stem cells under hypoxia secrete more microvesicles as a result of hypoxia (Zhang et al., 2012). Further supporting this notion, Kucharzewska et al. showed that glioblastoma-derived EVs are powerful inductors of angiogenesis *in vitro* through phenotypic regulation of endothelial cells: a number of growth factors and cytokines were induced in endothelial cells in order to boost the PI3K/AKT pathway as well as secrete several significant growth factors (Kucharzewska et al., 2013). Furthermore, hypoxic prostate cancer cells induced hypoxia-induced EVs that enhance the stemness and invasiveness of prostate cancer cells under normoxic conditions as well as enhancing the fibroblast phenotype in prostate stromal cells (Ramteke et al., 2015). Umezu and others found that Hypoxia-induced rapid tube formation in endothelial cells can be attributed to miRNA-135b in EVs released by hypoxia-resistant multiple myeloma cells (Umezu et al., 2014). Upregulation of HIF-1 has been shown to promote angiogenesis, and interestingly, miRNA-135b delivery by EVs inhibited FIH-1, a negative regulator of HIF-1. In recent times, Li et al. found that EVs extracted from hypoxic oral squamous cell carcinoma had an enhanced level of miR-21. Normoxic cells migrated and invaded after receiving this miRNA both *in vitro* and *in vivo* (Li et al., 2016). The aforementioned research offers compelling support for the observed biological consequences; they did not, however, obey commonly acknowledged protocols for EV isolation, which raises the likelihood of contamination. Also of interest is the fact that hypoxic tumor-derived EVs inhibit NK cell function by transporting TGFβ and microRNA-23a (Berchem et al., 2016). Therefore, hypoxic cancer cells appear to secrete EVs that are capable of promoting angiogenesis, metastasis, and immunosuppression (Figure 1).

In healthy tissues and organs as well as tumors, as an integral part of cellular communication, EVs are of great importance. It is the EV that serves as a carrier of nucleic acid, lipid, and protein cargo to facilitate intercellular communication, and its content varies from cell type to cell type and physiological state to physiological state (Balakrishnan et al., 2020). Studies of Hanson and van Niel have shown that ESCRT-dependent EV is biogenically produced and released by the coordinated action of ESCRT complex proteins (Hanson and Cashikar, 2012; van Niel et al., 2018). Proteins from the ESCRT-0 family contain ubiquitin-binding sites which are responsible for recognizing and sequestering ubiquitinylated cargo within late endosomes. Utilizing accessory proteins like ALG-2-interacting protein X (ALIX), ESCRT-0 proteins recruit ESCRT-I, II, and III complex proteins sequentially (Colombo et al., 2013; van Niel et al., 2018). ESCRT-I and -II complexes are essential for deforming the endosomal membrane and forming vesicular buds that hold cytosolic cargo, whereas vesicle breakage and ILVs’ release into MVBs involves ESCRT-III complex proteins (Mathieu et al., 2019). We’re starting to learn more about other vesicle-type pathways, like acidic sphingomyelinase (aSMase), which promotes m/lEV shed (Goñi and Alonso, 2006; Bianco et al., 2009). The outer leaflet of the membrane contains sphingomyelin, which is hydrolyzed by aSMase, which destabilizes it and lets m/lEVs shed (Tepper et al., 2000). Arf6 is another regulator that plays a role in m/lEV production and release by taking part in endosomal recycling (Muralidharan-Chari et al., 2009), and the small GTPase, RhoA (Li et al., 2012). Endosomes are the source of exosomes, and the final contents of exosomes are produced by their interactions with other intracellular vesicles and organelles. In addition to proteins, metabolites, amino acids, nucleic acids and lipids, they also contain other constituents, which can elaborate on their origins (Kalluri and LeBleu, 2020). Nucleic acids, lipids and proteins constitute the complex charges of EVs, which are composed of heterogeneous collections of membrane-bound carriers. The importance of EV release is becoming increasingly recognized in intercellular and even interorganismal communication, despite previously being viewed as merely a mechanism for removing non-functional cellular components. It is essential for the transmission of instructions between cells that EVs be stabilized and directed to certain cell types for delivery of their contents (Maas et al., 2017). Multiple processes have been proposed to contribute to EV formation. Both the exosome and the microvesicle, a vesicle that bubbles inward inside the endocytic system, require the induction of lipid curvature. One of the most well-studied steps in the formation of exosomes is the recruitment of the early endosomal ubiquitination signaling complex needed for transport (ESCRT) machinery to ubiquitinated proteins. VPS4, VTA-1 and Alix are accessory proteins which work in collaboration with the ESCRT machinery to bind and form intraluminal vesicles that contain future exosome cargoes (Christ et al., 2017). MVBs are formed when vesicles undergo inward budding and fission, which is induced by the spirals formed by the ESCRT-III complex (Lee I. H et al., 2015a; Chiaruttini et al., 2015; McCullough et al., 2015). Trajkovic et al. described that an alternate pathway is involved in the formation of exosomes. This involves the synthesis of ceramide, which is responsible for inducing vesicle curvature and budding (Trajkovic et al., 2008). Kajimoto’s team demonstrated that the transport of certain payloads in MVB ILVs is mostly dependent on ceramide synthesis (Kajimoto et al., 2013). Several mechanisms have been proposed for the biogenesis of exosomes, including the combination of tetraspanin and membrane proteins, such as the amyloid pre-melanosome protein and the tetraspanin receptor (Theos et al., 2006; van Niel et al., 2011). It is even more difficult to elucidate the molecular mechanisms that control the biogenesis of EVs that are derived directly from the plasma membrane (such as microvesicles). This process may be explained by the involvement of the ESCRT machinery, which is also involved in the promotion of ILV production in MVBs and viral budding. The ESCRT III proteins, which promote negative curvature, are recruited to facilitate the formation and fusion of exosomes and microvesicles (Lee I. H et al., 2015a; Chiaruttini et al., 2015; Christ et al., 2017). Former study has hown that the adaptor protein arresting domain containing protein 1 effectively recruits the ESCRT-I and VPS4 to the plasma membrane (Nabhan et al., 2012).

## 3 Diversity of RNA molecules in EVs of melanoma

Currently, extracellular RNAs that are aberrantly expressed in melanoma are mainly classified as miRNA, lncRNA and mRNA. MiRNAs (microRNAs) are a class of small endogenous non-coding single-stranded RNAs of 21-25 nt in length found in plants and animals that play a key role in the regulation of post-transcriptional gene expression in organisms. In humans, miRNAs are located in the introns of precursor mRNAs, a structural feature that allows mRNA genes and miRNAs in introns to co-transcribe, thus playing an important role in human growth, development and disease (Hill and Tran, 2021). lncRNAs (long non-coding RNAs) are DNA transcripts greater than 200 nucleotides in length that are not involved in protein coding processes. Most lncRNAs have been identified as key regulators of transcription and translation and can play specific roles through two different pathways: protein and microRNA networks (Bridges et al., 2021). Messenger RNA (mRNA) is a transcription of DNA that carries the corresponding genetic information to provide the information needed for further translation into proteins. As a therapeutic target, it provides a new approach to the treatment of melanoma (van Dülmen and Rentmeister, 2020).

### 3.1 MiRNAs in EVs of melanoma and their relative signal pathway of melanoma

We list miRNAs in EVs which be essential in melanoma development in Table 1 (Pansky et al., 2000; Segura et al., 2009; Shull et al., 2012; Dror et al., 2016; Felicetti et al., 2016; Chowdhury et al., 2018; House et al., 2018; Huber et al., 2018; Shu et al., 2018; Svedman et al., 2018; Unterer et al., 2018; Lee et al., 2019; Li et al., 2019; Luan et al., 2021; Lucianò et al., 2021; Torii et al., 2021; Gerloff et al., 2022; Hathaway-Schrader et al., 2022). MiR-222 is a miRNA which confirmed to be modulated by PI3K/AKT signaling (Felicetti et al., 2016; Santos and Souza, 2019). Given the correlation between miR-222 and these oncogenic pathways, circulating miR-222 has been investigated as a potential tumor biomarker (Calderaro et al., 2014; Chen et al., 2014; Teixeira et al., 2014; Lee J. C et al., 2015b). Federica et al. found that miR-222 induced upregulation of PI3K/p85β subunits in melanoma cells and exosomes. In the subsequent study, they demonstrated that miR-222 and exosomes transfer to recipient cells had sufficient effect on regulating the PI3K/AKT pathway after internalization. Furthermore, they demonstrated that secreted exosomes reduced p27Kip1 (a direct target of miR-222), which increased melanoma invasion and chemotacticization. Using their results, it was determined that miR-222 can be transferred between cells as content of melanoma exosome transporters, thus promoting melanoma by activating several molecules, including the PI3K/AKT pathway as well as the MAPK axis. Thus, miR-222 has been demonstrated to function as a biomarker for melanoma diagnosis and prognosis (Garofalo et al., 2012; Felicetti et al., 2016).

**TABLE 1:** The roles of miRNAs in EVs of melanoma.

RNA ID	Effect	Target Site	EV origin	Reference
miR-222	Benefit tumor progression	p27Kip1/CDKN1B, c-KIT,c-FOS	Melanoma EVs	Hill and Tran (2021)
let-7g-5p	Related to melanoma metastasis	MAPK	Patient’s plasma	David et al. (2016)
miRNA-34a	Inhibition of the proliferation of melanoma cell	β-catenin	Patient’s plasma	Sarvaiya et al. (2013)
miR-211	Inhibition of the expression of an ERK5 pathway-specific phosphatase, DUSP6	IGF2R	Melanosome	Mikuła-Pietrasik et al. (2015)
miR-21	Enhanced the invasive potential of melanoma cell lines	PTEN	Melanoma	Valadi et al. (2007)
miR-205, miR-182, miR-200c	Inhibition the distant metastasis of melanoma	E2F1, E2F5	Melanoma	Zhou et al. (2020)
miR-709, miR-2137	Regulation of the T-cell function	PD-L1	Melanoma EVs	Meyers et al. (2018)
miR-494	Inhibition of the tumor growth and metastasis		Melanoma EVs	Chen G. et al. (2018b)
miR-106b-5p	Activation of the ERK pathway	EphA4	Melanoma EVs	Poggio et al. (2019)
miR-146a, miR-155, miR-125b, miR-100, miR-125a, miR-99b	Transform the myeloid cells into myeloid-derived inhibitory cells	CTLA-4, PD-1	Melanoma EVs	Yang et al. (2018)
miRNA-1246	promoting the expression of IL-6	STAT3, Akt	Melanoma EVs	Lennon et al. (2019)
miR-92b-3p, miR-182-5p, miR-183-5p	Promoting the angiogenesis of melanoma	FOXO1、CMTM7、VASH1、THBS2、SOCS3、PIK3R1、TSP-1、CTGF	Melanoma EVs	Murillo et al. (2019)
miR-155, miR210	Benefit tumor progression	OXOPHOS	Melanoma EVs	Wei et al. (2013)
miR-214-3p, miR-199a-3p, miR-155-5p	Promoting the metastasis of melanoma		Melanoma EVs	107
miR-214, miR-199a	Related to the progression of melanoma	TWIST-	Melanoma EVs	107

MiR-155 can upregulate glucose metabolism, leading to increased glycolysis (Kim et al., 2018), and upregulation of miR-210 can reduce OXPHOS (oxidative phosphorylation) under non-hypoxic conditions (Grosso et al., 2013). Shu et al. found that exosomes from six melanoma cell lines contained miR-155 and miR-210. Furthermore, the researchers demonstrated that human adult skin fibroblasts (HADF) have reprogrammed their metabolic functions due to the presence of these metabolic regulatory miRNAs by transfecting human melanoma-derived exosomes (HMEX). A red fluorescent protein RNA marker was observed to show that exosomes can transport RNA cargo into the HADF. They also examined glycolysis levels in exosomes using the same technique and found that they had significantly decreased following the suppression of miR-155/miR-130 expression in HADF and the reversal of OXPHOS downregulation caused by HMEX within HADF. TF-HMEX-NC showed a significant 61% reduction in basal respiration, 39.3% reduction in maximal respiration, and ATP production was reduced by 36.5% (Mugoni et al., 2022). These results suggested that miR-155 and miR-210 are delivered to the receptor HADF via melanoma exosomes, affecting HADF’s metabolic preferences by upregulating glycolysis and downregulating OXPHOS in melanoma, thereby favouring a pre-metastatic microenvironment (Shu et al., 2018).

**FIGURE 2:**
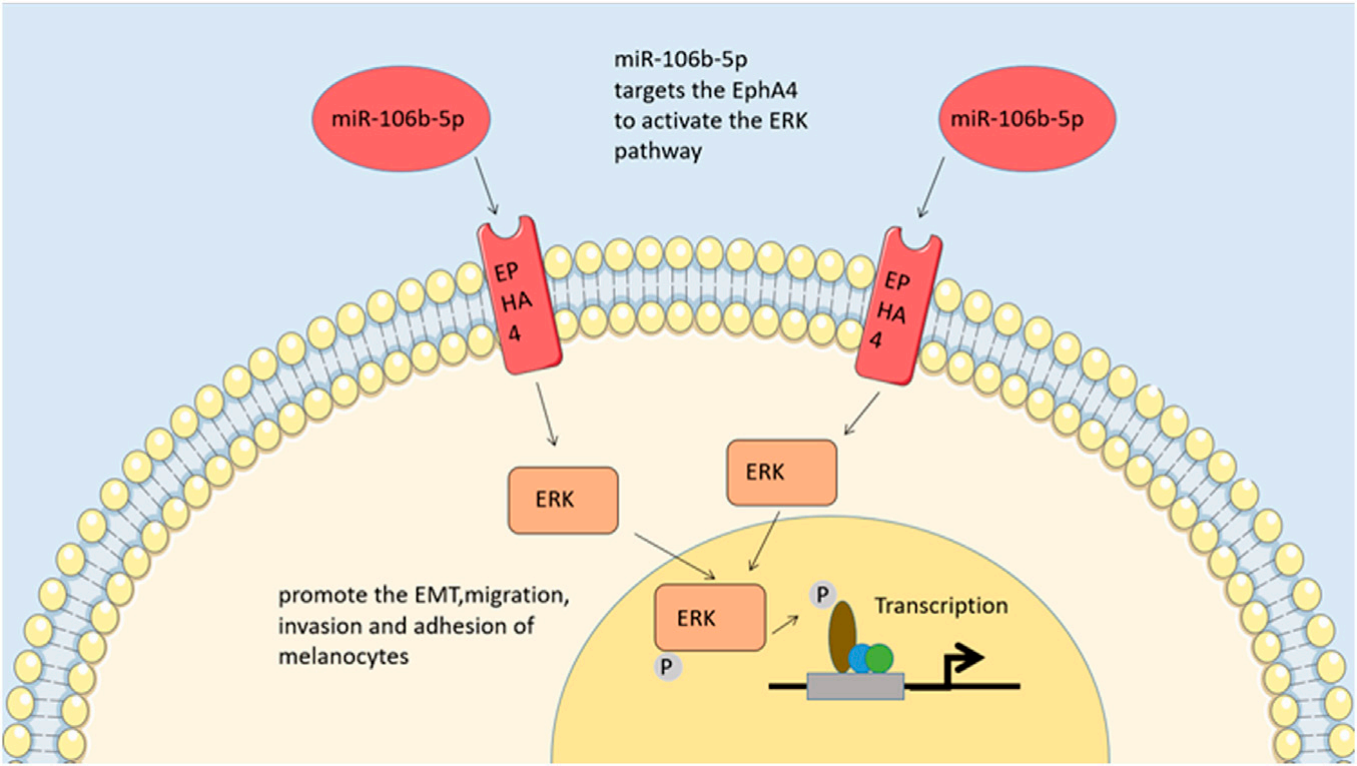
ERK pathway. By regulating EphA4, MiR-106b-5p activates the pathway of ERK to promote the epithelial-tomesenchymal transitions, migration, invasion and adhesion of melanocytes.

MiR-106b-5p has been shown in many investigations to be disproportionately represented in melanoma-derived exosomes and exosome-mediated melanoma cell transfers (Tengda et al., 2018). Melanocyte migration, invasion, and adhesion are promoted by miR-106b-5p found in exosomes. The effect of this compound is achieved through activating the ERK pathway by targeting EphA4 (Luan et al., 2021). A high metastatic propensity of melanoma is associated with the expression of epigenetic transcription factor, which are also expressed by normal melanocytes (Caramel et al., 2013). In multiple types of human tumors, EphA4 functions as an oncogene and tumor suppressor, contributing to the progression of erythropoietin hepatocellular carcinoma (Fukai et al., 2008; Saintigny et al., 2012). By blocking ERK activation, it prevents melanoma cells from undergoing EMT and subsequently spreading (Hua et al., 2018). Melanoma metastasis *in vivo* is facilitated by exosomal miR-106b-5p activation of the ERK pathway and promotes EMT in melanocytes. Melanoma patients may benefit from exosomal miR-106b-5p, a diagnostic indicator and potential therapeutic target derived from melanoma cells (Luan et al., 2021). Through studying the molecular biology of tumor-derived exosomes containing EMT regulators, novel diagnostics and treatment approaches for melanoma may be identified (Figure 2).

MiR-182 is overexpressed in melanoma cells compared to melanocytes, and silencing it leads to cell death. It decreases fibronectin invasion in both SK-MEL-19 and A375 cells after being effectively silenced, indicating that miR-182 may provide a survival benefit to melanoma cells or be necessary for them to preserve their oncogenic properties. MiR-182 overexpression did not enhance the oncogenicity of existing melanoma cells, but did strengthen the immortalization of normal human melanocytes. Melanoma cells are stimulated to migrate and invade, enhancing their capacity to metastasize and penetrate the extracellular matrix. MiR-182 can also enhance the extravasation ability of melanoma cell, also known as distant seeding. Additionally, miR-182 expression was shown to be inversely correlated with MITF and FOXO3 levels in metastatic melanoma. It directly targets FOXO3, which regulates the migration, apoptosis and cell cycle in tumorigenesis. Melanoma cells that contain miR-182 and its downstream effectors may provide prognostic information and also be therapeutic targets in the future (Segura et al., 2009).

### 3.2 LncRNAs in EVs of melanoma

Subnuclear paraspeckle bodies rely on NEAT1, a lncRNA that serves as a structural backbone (Machitani et al., 2020). Choroidal neo-vascularization and M2 polarization, both crucial steps in melanoma’s development and maintenance, are significantly upregulated in melanoma (Bardi et al., 2018; Zhang et al., 2020). Cancer is influenced by the expression of mRNAs by non-coding RNAs, known as non-coding RNAs, and miRNAs by lncRNAs (Fathullahzadeh et al., 2016; Chen L. et al., 2018a; Sun et al., 2019). An EV-carrying NEAT1 plays a crucial role in melanoma progression by binding miR-374a-5p to promote the recruitment of IQGAP1, thereby enhancing the expression of LGR4 and promoting the polarization of macrophage M2 ^79^. Macrophages can internalize BMSC-EVs carrying NEAT1 and increase LGR4 expression by binding to miR-374a-5p in a competitive manner to recruit IQGAP1 by causing miR-374a-5p to bind to IQGAP. As a consequence, macrophage M2 becomes polarized, which promotes cell proliferation, angiogenesis, and invasion of melanoma cells as well as inhibiting their apoptosis. According to the data presented above, NEAT1 may represent a novel clinical treatment target for melanoma (Yang et al., 2022).

Other ncRNAs with documented functions in ribonucleoprotein complexes like tRNA and rRNA processing, such as RNase P and RNase MRP, were also shown to be highly represented in melanoma iEVs. By binding to TERC (the RNA component of telomerase) and activating the enzyme, Pop proteins may be involved in the assembly of six of the ten protein subunits that make up the Pop and MRP RNAses of telomerase holoenzyme. They also participate in the reactivation of telomerase in zebrafish melanoma, which disrupts the equilibrium between the RNA and protein components of P and MRP RNases. After inflammation brought on by IEVs, macrophages that have taken in melanoma iEVs show an increase in IRG expression, suggesting that this process activates RNA sensing pathways. More research into the primary inflammatory signals carried by melanoma iEVs may be necessary for the development of techniques to regulate inflammatory responses, the repercussions of which on tumor growth and metastasis are currently unknown. When injected into larvae, melanoma iEVs cause inflammation and an upregulation of interferon-responsive genes; this action can be mimicked by infusing MRP- or P-RNAs into the bloodstream. This demonstrates that zebrafish melanoma iEVs include MRP- and P-RNAs, which can induce inflammation in innate immune cells (Biagini et al., 2022).

In addition to propagating and migrating cancer cells, melanoma-derived exosomes reprogrammed fibroblasts into tumor-promoting cancer-associated fibroblasts (CAFs) (Hu and Hu, 2019). CAFs are commonly known as fibroblasts, which are connective tissue cells that can be activated in tumors. Schoeppe et al. found that CAFs were interact with cancer cells in tumor microenvironment, promoting tumor metastasis and progression (Schoepp et al., 2017). B16F0-Exo-mediated transformation of fibroblasts into CAFs as a result of high Gm26809 expression in melanoma cells (B16F0). NIH/3T3 cells were markedly migrated through Gm26809, thereby converting fibroblasts into CAFs when Gm26809 was overexpressed. Alternatively, knockdown of Gm26809 resulted in a reduction of the exosomes secreted by Gm26809, which hindered the exosome-induced NF-CAF transition, thereby inhibiting the proliferation and migration of melanoma cells mediated by exosomes, indicating that exosome Gm26809 could provide a potential spot for inhibiting the progression of melanoma (Hu and Hu, 2019).

### 3.3 MRNA in EVs of melanoma

There is a relationship between cutaneous melanoma and lymphatic metastasis, and the EV secreted by melanoma is also spread by lymphatic channels (García-Silva et al., 2021). The inflammation-associated mRNA content of melanoma EV differs from that of primary melanocytic EV. CXCL1, CXCL2 and CXCL8 mRNAs were determined to be upregulated (Bardi et al., 2019). CXCL1 is involved in a wide variety of processes, including inflammation, angiogenesis, cancer, and wound healing. Recent years have shown conclusively the significance of chemokines in cell transformation, tumor development, homeostasis, and metastasis. CXCL1 expression is not intrinsically driven in normal human epidermal melanocytes (NHEM) or normal retinal pigment epithelium (RPE). To counteract this, IL-1, lipopolysaccharide, and tumor necrosis factor alpha (TNF-α) can all stimulate CXCL1 expression. A 306-bp minimal promoter is primarily responsible for generating transcription of the CXCL1 gene. The four cis-combining elements containing the minimal promoter include the TTATA cassette, the NF-κB binding site, the AT-rich high mobility group protein I (HMGI) Y) binding element nested within the NF-κB site, the direct upstream field and the GC-rich SP1 binding site. NF-κB is a group of proteins whose expression is controlled by transcriptional, translational or post-transcriptional mechanisms. Constitutive production of chemokines and disruption of the NF-κB transcriptional machinery are hypothesized to play pivotal roles in the earliest stages of cancer formation. DNA-binding proteins are a family of related proteins that play an important role in controlling cell proliferation, differentiation, and apoptosis by modulating the expression of promoter-driven and anti-apoptotic genes. Rel/NF-B is a family of genes involved in regulating cell growth and carcinogenesis. In unstimulated, untransformed cells, NF-κB is isolated in the cytoplasm and forms a complex with IκB, which subsequently masks its nuclear localization signal, thereby preventing nuclear translocation of NF-κB (Dhawan and Richmond, 2002). It is therefore relevant to determine the grade of melanoma by measuring the levels of CXCL1 mRNA in melanocytes to determine whether overexpression of CXCL1 in melanocytes can cause tumor formation and metastasis.

**FIGURE 3:**
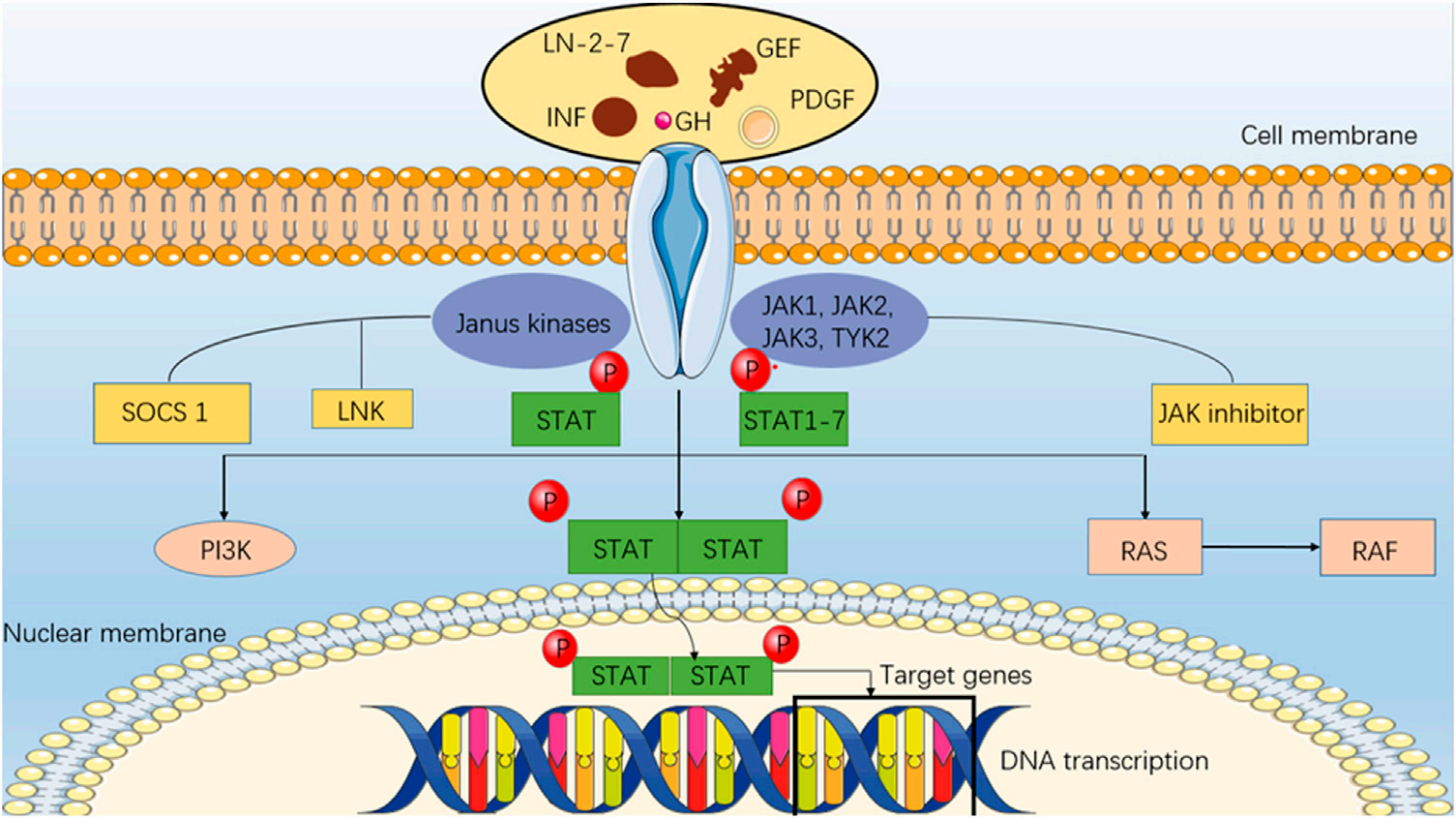
JAK/STAT pathway. An intracellular pathway (JAK/STAT) consists of a class of intracellular molecules (Janus Kinase) that respond to upstream receptor signals and the activated JAK catalyzes tyrosine phosphorylation of the receptor by rapidly recruiting to the receptor and activating it. In addition to binding to the receptor molecule, phosphorylated tyrosine on it is a binding site for signaling molecules, such as SH2, that bind to the receptor and phosphorylate tyrosine, forming a dimer that enters the nucleus, and STAT (signal transducer and activator of transcription). Regulation of the JAK/STAT pathway is mediated by interferon binding and activation of its receptor.

CXCL1 and CXCL2 share 90% identity in amino acid sequence and have the same receptor CXCR2 (Shi et al., 2018). By promoting recruitment and expansion of CD11b+ myeloid suppressor cells to the tumor, they released by melanoma cells can enhance melanoma survival. In tumors, CD11b+ marrow cells are also an extra source of CXCL1 and CXCL2 production (Shi et al., 2018). CXCL-8 (interleukin 8 or IL-8), which is constitutively secreted by melanoma cells (Singh et al., 1994), is expressed on melanoma and endothelial cells by two high-affinity receptors for CXCL-8, CXCR1 and CXCR2 (Li et al., 2002), and activation of CXCL-8 signaling opens up cell proliferation, apoptosis inhibition, cell cycle control and cytoskeletal dynamics involving various downstream pathways and transcription factors. Wu et al. found that CXCL-8 and the expression of its receptors are positively correlated with the aggressiveness of melanoma. Melanoma patients may benefit from treatments that block CXCL-8 signaling or its receptor, according to preliminary research (Wu et al., 2012). Furthermore, CXCL-8 was also shown to contribute significantly in the regulation of the JAK/STAT pathway. According to studies, the melanoma cells in tissues exhibit marked heterogeneity compared to adjacent normal tissues, most of which exhibit elliptical appearances that range in shape and size. Compact arrangement, distinct nuclei, mitotic appearance, large cytoplasm, diffuse distribution, and high yield of intracellular and intercellular melanin granules, showing the characteristics of melanocytes in a biological sense. In the following gene expression experiment, E-calponin expression was drastically lower in melanoma tissue as compared to the surrounding normal tissue, while CXCL8, JAK2, STAT3, vimentin, and N-calcineurin expression was significantly increased. There may be a higher expression of CXCL8 and activation of the JAK-STAT signaling pathway present in cutaneous melanoma, in addition to the presence of epithelial-mesenchymal transition (EMT). Activation of the JAK-STAT signaling pathway inhibits the proliferation of cancer cells, whereas silent expression of CXCL8 promotes the proliferation of cancer cells. It was further noted that each group had a different apoptotic status. Apoptosis of cancer cells can be promoted by inhibiting the expression of CXCL8, while apoptosis can be blocked by activating the JAK-STAT pathway. It can be concluded from these studies that silencing the expression of CXCL8 in human cutaneous melanoma cells may inhibit EMT and cell proliferation while promoting apoptosis through the suppression of the development of the JAK-STAT signaling pathway (Hu et al., 2020). If we can clarify the role of CXCL8 and JAK-STAT signaling pathways in EMT and apoptosis, it will have important implications for the diagnosis, treatment and screening of cutaneous melanoma. Especially if we discover signal transduction substrates, activators, and inhibitors. As part of its paracrine immunosuppressive function, IL-8 is also produced by tumors, which promotes cancer survival. As a result of CXCL8, tumor-associated neutrophils (TAN) are recruited, which recruit pro-tumor regulatory T-cell (Tregs) and suppress the anti-tumor immune function of adaptive T-cell. CXCL8 also recruits medically important stromal cells (MSCs), which suppress the anti-tumor immune function of adaptive T-cell (David et al., 2016). Cells also produce CXCL8/IL-8 expression during senescence or cell growth arrest (Sarvaiya et al., 2013). Human peritoneal mesothelial cells that have reached their end of life produce conditioned media that stimulates the growth of numerous tumor cell lines by releasing CXCL1 and CXCL8 (Mikuła-Pietrasik et al., 2015). Considering that sEV might be present in conditioned medium and that sEV can transport translatable mRNA to its intended recipient cells (Valadi et al., 2007), perhaps the inflammatory crosstalk mRNA detected in melanoma sEV is functionally relevant. The present study hypothesizes that tumor sEV CXCL8 mRNA levels may be used to monitor recurrence risk during remission and monitor tumor senescence (Figure 3). As shown in Table 2, a summary of mRNA expression is found in the EVs of melanoma.

**TABLE 2:** The roles of mRNAs in EVs of melanoma.

RNA ID	Expression	Effect	EV origin	Reference
NEAT1	up	Promoting the polarization of macrophage M2 and thus acclerating the progression of melanoma	Melanoma EVs	Calderaro et al. (2014)
RNAse P and RNAse MRP	up	Activation of telomerase	Melanoma EVs	Lee J. C et al. (2015b)
Gm26809	up	Reprogramming fibroblasts into tumor-promoting CAFs, thereby promoting melanoma cell proliferation and migration	B16F0 cells	Chen et al. (2014)
CXCL1	up	Promoting the proliferation of melanoma	Melanoma cells, CD11b + bone marrow cells	Caramel et al. (2013), Grosso et al. (2013)
CXCL2	up	Promoting the proliferation of melanoma	Melanoma cells, CD11b + bone marrow cells	Caramel et al. (2013), Grosso et al. (2013)
CXCL8	up	Mediates tactile migration and MMP 2 production in melanoma cells, further promoting extracellular matrix degradation and melanoma migration	Macrophages, epithelial cells, and auto circulation	Grosso et al. (2013), Machitani et al. (2020)
BCL-XL	down	Inhibition the apoptosis of melanoma cells	melanoma cell	Welsh et al. (2020)
STAT-3	Up	Induction of anti-apoptotic, angiogenesis, immunosuppression, and metastatic gene expression profiles favors melanoma growth and survival	melanoma cell	Tian et al. (2018)
HLA-C	down	Reducing immune surveillance and promoting immune escape of melanoma	major histocompatibility complex, MHC	Geeurickx et al. (2019)
GBP1	down	Promoting endothelial cell proliferation	melanoma cell	Cheng et al. (2021)

## 4 Study on exRNAs and tumor microenvironment in melanoma

### 4.1 High-throughput nano-bio–chip integrated system for liquid biopsy (HNCIB)

Recent developments suggested the capability to capture and visualize single-EVs as well as to simultaneously detect EV internal surfaces and cargo components using an integrated HNCIB system. So far, there has been no agreed-upon method for measuring individual EVs, despite the fact that this capacity would be of enormous use to the field of EV research as a whole if it existed. Due to the excellent signal-to-noise ratio of this system, it was capable of detecting individual EVs and high-resolution detections. Using a training set, Zhou et al. developed, trained, and improved an algorithm based on deep learning for image screening. This algorithm enables intelligent screening of fluorescence images derived from experimental data. EV particle fluorescence spots are also distinguished from those caused by contaminants or noise using additional modules included in the algorithm, which contribute to an improved signal quality. With the goal of increasing the biological signal-to-noise ratio of the HNCIB, several improvements were also made in the study. The nano biochip was optimized for EV capture, was improved for washing efficiency in order to eliminate impurities, was enhanced for specificity, and the fluorescent antibody was fine tuned for optimal performance (Zhou et al., 2020).

However, considerable work has to be done before miRNAs, mRNA, and proteins stored in/on EVs are considered viable cancer biomarkers, despite the impressive progress made in EV-based liquid biopsy. EV liquid biopsy may be able to address these issues as a result of the advantages and unique capabilities of the HNCIB. The immunohistochemistry identification of PD-L1 expression in tumor tissue samples is a frequent diagnostic technique for immunotherapy of advanced disease, particularly non-small cell lung cancer. There’s no way to test for PD-L1 expression multiple times in lung tissue because PD-L1 expression is dynamic in cancer cells (Meyers et al., 2018). It is therefore extremely difficult to track the changes in PD-L1 expression during immunotherapy. The use of liquid biopsy, on the other hand, offer an alternative to the utilization of immunohistochemistry for PD-L1. By remotely inhibiting PD-1 on CD8 T-cell, exosomal PD-L1 can help cancer cells elude immune system responses (Chen G. et al., 2018b; Poggio et al., 2019). EVs from healthy donors and patients with LUAD were analyzed by HNCIB for changes in PD-L1 mRNA and protein. While PD-L1 miRNA and protein were measured in EVs from lung cancer, other miRNA, mRNA, and proteins could be measured in EVs from patients with other types of cancer with different antibodies or probes.

EV proteins and mRNA can be detected simultaneously using the newly developed HNCIB system. The method has a high signal-to-noise ratio, high sensitivity, and high specificity for recognizing surface and cargo biomarkers of EVs since it uses machine learning in conjunction with image processing. In addition to being able to do high-throughput analysis, it also has the potential to give enhanced accuracy with the use of machine learning. A potential clinical application of the technology may enhance cancer care and contribute to advancements in liquid biopsy. Athough HNCIB has only been studied in clinical practice for patients with LUAD, it will be valuable in many aspects of cancer care, such as early cancer identification, precision therapy, evaluation of treatment response, and early detection of recurrences (Yang et al., 2018).

### 4.2 The MPApass programme paves the way for standardized reporting of bead-based assays and allows for integrated multiplex, multidimensional analysis of EV repertoires

Using stitched multiplex analysis post-acquisition analysis (MPApass), Welsh et al. showed how millisons of protein combinations on EVs can be analyzed ergonomically on EVs. With the help of this tool, assays involving single EVs and highly sensitive markers can be identified (Lennon et al., 2019). In addition, this approach may allow the identification of markers for further large-scale tests, including RNA sequencing, that could be used to isolate targeted subsets. In complex fluids, the deconvolution algorithms previously developed for RNA sequencing can provide insights into EV derivation by stratifying RNA sequencing data by protein subsets (Murillo et al., 2019). In order to maintain the current ERCC exRNA Atlas, MPAPASS was designed for curation of subsequent datasets. An analysis and reporting toolkit were developed as part of the software. Notably, MPAPASS provides these tools for bead-based EV analysis; on the other hand, FCMPASS software uses the MIFlowCyt-EV reporting framework in order to provide a standardized tool for single EV flow cytometry analysis (Welsh et al., 2020). Fluorescence calibration '. fcs’ file can be calibrated using FCMPASS before being imported into MPAPASS for analysis. By adopting these standardized reporting and analytic techniques, EV repositories will become open, repeatable, and integrated, paving the way for innovative ways of EV characterization and the determination of EV subsets (Tian et al., 2018).

Standardization and reproducibility of these tests in scientific research are key factors in the analysis of EV repertoires by multiplex assays. Standardization and quality assurance can be improved in several ways: 1) to assist in the standardization of batches and types of beads, it is necessary to quantify the binding capacity of each bead; 2) plan for the creation of pathology-specific monoclonal antibodies and matching monoclonal beads to create tailored multiplex arrays for validation testing and pull down; 3) the possibility of calibrating and comparing data using Molecules of Equivalent Soluble Fluorophore standards; 4) creating a standard set of reporting requirements according to current reporting standards for single-EV flow cytometry (Tian et al., 2018).

Based on the results of this research, stitched multiplex analysis in conjunction with MPAPASS software is an effective method for generating, evaluating, and analyzing data from a large number of marker combinations. The quantitative single-EV approaches or bulk EV subset analysis techniques that may be derived from this work are also outstanding. This approach has a number of limitations, most notably the limited number of markers it can screen simultaneously on EV-containing samples, and its semiquantitative nature (Welsh et al., 2020). As part of a systematic multiplex-to-single EV process, stitched multiplex analyses using MPAPASS provide a means for EV repertoire analysis, allowing for the further quantification of subgroups of EVs with known pathologies. The use of MPAPASS will make it easier to discover new EV biomarkers and investigate the roles of EV subsets in health and disease (Tian et al., 2018; Welsh et al., 2020).

### 4.3 FO‐SPR (fiber optic surface plasmon resonance) biosensor

According to Yildizhan et al., EVs may be specifically and sensitively detected using a FO-SPR biosensor in a variety of complicated matrices, such as blood plasma and cell culture medium. Their analysis was conducted using rEV as a biological reference material for specific FOSPR detection of EV (Geeurickx et al., 2019). In order to achieve repeatable immobilization of distinct capture antibodies on the FOSPR sensor surface and to detect EVs specifically, many components of the bioassay were improved. Supporting this specificity was the observation that an SPR and GFP-mediated microscopy signal could only be detected when an EV-specific antibody, and not a non-specific anti-IgG antibody, was used on the FO-SPR surface. With six different combinations of EV-specific antibodies used to capture and detect EVs, the scientists upgraded the label-free bioassay to a sandwich bioassay for further improvement. Using a biotinylated detecting antibody and an anti-biotin antibody-functionalized AuNPs, the researchers successfully implemented an increase in strength of the signal in two stages technique on the FO-SPR device. It was important for EV detection to be performed at a low pH6 while still retaining AuNPs in a pH 7.4 PBS buffer step with this approach. As a result, the approach not only produced significant SPR shifts, but also assisted in EV detection at a pH of 6. EV concentrations in human plasma and cancer patients’ plasma are 103-fold and 104-fold lower, respectively, than the expected physiological concentrations. This level of sensitivity is indicative of the great potential of the well-established FO-SPR technique for EV detection and analysis and may be especially relevant when the goal is using specific biomarkers to detect various subpopulations of EVs, rather than just the total number of EVs in patients’ samples. Subsequently, robustness and specificity of the created sandwich bioassay were evaluated by identifying three various EV kinds in two various complicated matrices (Geeurickx et al., 2019).

The research results suggested that it is necessary to start the development of EV detection methods early in the process by employing pre-tested normative materials such as rEVs. Additionally, the conclusion obtained demonstrated: 1) The FO-SPR device has exceptional sensitivity for detecting and counting EV, 2) in highly matrix-rich environments like cell-culture media and plasma, there is the possibility for direct measurement, and 3) the presence of a biomarker for the disease in question, EVs originating from cancer cells can be distinguished from those originating from healthy cells. This, in addition to other inherent advantages like real-time monitoring, rapid reaction times, parallel measuring capabilities, and simplicity of operation, suggests it could be used as a benchtop EV biosensor platform.

## 5 Conclusion and prospects

While previous review has summarized extracellular vesicles in melanoma (Cheng et al., 2021), this review focuses more on extracellular RNA in melanoma, with a comprehensive overview of lncRNA, cirRNA, and mRNA in addition to miRNA. Besides, we also analyse the typical signaling pathways which extracellular RNA involved in and the most up-to-date tumor immunotherapy means targeting exRNAs.

Researchers have been exploring into the causes and consequences of immunotherapy resistance in order to discover a way forward. Current consensus on the most effective population-based screening criteria for PD-1 benefit is summarized as follows: high tumor mutational load (TMB), high microsatellite instability (MSI-H), defective mismatch repair (dMMR) and high PD-L1 expression with tumor infiltrating lymphocytes (TIL). There have been some clinical trials that examined combinations of immune checkpoint inhibitors with targeted agents and demonstrated some clinical benefit in addition to the immunotherapy summarized above. Combining immune checkpoint inhibitors with immune factor antagonists (CD40, CD137, CD134, CD357) has demonstrated some synergistic benefits in the treatment of advanced melanoma in clinical studies (Wei et al., 2013). In addition, it has been shown that the use of many treatment modalities, including as radiation, chemotherapy, surgery, and cancer vaccines, may synergize with immunotherapy to increase antitumor effectiveness by modulating the activation of immune factors in the tumor microenvironment (Chowdhury et al., 2018).

Drug combination therapy for melanoma is currently being researched. Better comprehension of the molecular mechanisms underlying the undesirable consequences of combination therapy is crucial for increasing the efficacy of tumor immunotherapy. When it comes to treating melanoma and other malignancies, immunotherapy appears to usher in a whole new era. Despite the fact that individuals with metastatic melanoma frequently exhibit primary or acquired resistance to immunotherapy, this treatment option remains promising. Patients with advanced melanoma, however, continue to benefit from this treatment. Research has demonstrated that it allows patients to live longer and maintain a high standard of living. Understanding how individual tumors evade the immune system is one of the key components of effective immunotherapy. In order to achieve the best response and improve overall outcomes, it is necessary to improve our understanding of these mechanisms and identify immune markers specifically associated with each patient.

Consequently, further investigation into the mechanism of multiple exRNAs in the melanoma extracellular capsule on melanoma development and metastasis is necessary, since this might be immensely beneficial in melanoma treatment. For example, lncRNAs can be inactivated by small interfering RNAs and/or antisense oligonucleotides (ASO), which are potential therapeutic targets. CXCL8 and JAK-STAT signaling pathways play a pivotal role in EMT and apoptosis in cutaneous melanoma. Melanoma of the skin can be diagnosed, treated, and drug-screened by understanding these pathways. In conclusion, as pathological mechanisms and other studies continue to be investigated, a broader range of therapeutic approaches will be developed, and the outcomes will undoubtedly acquire more extraordianry and become a boon for melanoma patients.
